# New perspectives for the treatment of the patient with sepsis

**DOI:** 10.1590/1518-8345.0000.3082

**Published:** 2019-01-14

**Authors:** Evelin Capellari Cárnio

**Affiliations:** 1 Universidade de São Paulo, Escola de Enfermagem de Ribeirão Preto, PAHO/WHO Collaborating Centre for Nursing Research Development, Ribeirão Preto, SP, Brazil.

**Figure:**
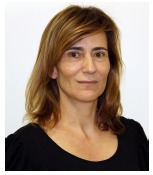

Sepsis represents 20% of admissions in non-cardiologic intensive care units (ICU) and has a high mortality rate. Even with the advances in antimicrobial and vasopressor therapy, mortality remains high. Thus, early diagnosis of sepsis is necessary to avoid septic shock, which is associated with a mortality rate of 40% or more^1^.

In 2016, the Society of Critical Care Medicine (SCCM) and the European Society of Intensive Care Medicine (ESICM) published new definitions, simplifying the nomenclature of sepsis, defining it as life-threatening organic dysfunction caused by an unregulated response to infection. Septic shock is when sepsis occurs with the presence of hypotension, requiring vasopressor therapy to maintain mean blood pressure (MBP) ≥ 65 mmHg associate with lactate ≥ 2 mmol/L, after adequate fluid resuscitation^2^.

In response to the lack of improvement in the mortality of patients with sepsis, the same societies created the campaign Surviving Sepsis, which provided guidelines for the treatment of the disease worldwide. These guidelines were reviewed in May 2018 and underwent some changes. The main alteration was the proposal of the hour-1 bundle^3^. The sepsis management bundle comprises a selected set of care actions that, when implemented as a group, can affect the clinical outcome and simplify the complex care process of these patients. Prior to the alterations, the campaign included two care bundles with the purpose of reducing mortality. The first was called 3-hour bundle and included measuring lactate levels, obtaining blood cultures prior to administration of antibiotics, administering broad-spectrum antibiotics and administering 30 mL/kg crystalloid in case of hypotension or lactate greater than or equal to 4 mmol/L. The purpose of this bundle was to prevent tissue hypoxia and hypoperfusion and, at the same time, to institute early antimicrobial therapy. The 6-hour bundle included the use of vasopressor therapy to maintain MBP greater than or equal to 65 mmHg, in the case of persistent hypotension, even after fluid replacement, with blood pressure less than 65 mmHg or lactate greater than or equal to 4 mmol/L.

The most important change in this review was that the 3-hour and the 6-hour bundles were combined into a single hour-1 bundle, with the purpose of starting interventions as quickly as possible. This measure favors the care provided at the bedside of patients, with therapy that begins immediately, especially for those with hypotension, instead of waiting longer and subsequently having to deal with more complex resuscitation measures for longer periods.

This new bundle includes:

Measuring serum lactate level. Increases in this concentration suggest the possibility of tissue hypoxia and accelerated aerobic glycolysis driven by excessive beta-adrenergic stimulation, which may be associated with worse prognosis.

Obtaining blood cultures prior to administration of antibiotics. The collection of blood cultures is an indispensable step in the management of sepsis. Cultures must be obtained before antibiotic administration, considering that the sterilization of cultures may occur.

Administering broad-spectrum antibiotics. Broad-spectrum antibiotic therapy should be initiated with one or more intravenous antimicrobials to cover all possible pathogens. It must begin immediately after collecting the blood.

Begin fluid resuscitation with 30 mL/kg crystalloid for hypotension or lactate greater than or equal to 4 mmol/L. Early fluid resuscitation is crucial for the stabilization of sepsis-induced tissue hypoperfusion or septic shock

Beginning vasopressor therapy if the patient presents hypotension during or after fluid resuscitation, to maintain mean blood pressure greater than 65 mmHg. If the hypotension is not controlled after the initial fluid resuscitation, then vasopressors should be commenced within the first hour to achieve MBP ≥ 65 mmHg.

That’s the main change in the bundles. Previously, vasopressor therapy was commenced only in the 6-hour bundle. However, urgent restoration of perfusion pressure is essential for the proper functioning of the vital organs and a key part of resuscitation that should not be postponed.

The bundles have evolved; however, the essence of the campaign Surviving Sepsis remains the same, that is, identification and care must be immediate in order to improve the prognosis. Despite criticisms, the care bundles for patients with sepsis and septic shock are supported in the literature and an individual clinical analysis of the patient at the bedside is necessary. In this sense, the nursing team, that remains close to the patient for longer periods, can assist in the recognition and early diagnosis of the disease. Through the identification of the basic needs affected, this professional can contribute to the multi-disciplinary team, evaluating the patient and using appropriate therapies that can contribute to the best prognosis. Thus, it is necessary to train nurses who work in the ICU to acquire skills and to have a protocol-oriented approach in order to start effective and early therapy. The guidelines in the campaign Surviving Sepsis offer a solid structure for the management of the disease, empowering nurses to make a difference in patient care. In addition, this professional can provide important data that should be considered in the formulation of these care bundles.
